# Chronic Lead Exposure Results in Auditory Deficits and Disruption of Hair Cells in Postweaning Rats

**DOI:** 10.1155/2019/4289169

**Published:** 2019-05-14

**Authors:** Shou-Sen Hu, Shi-Zhong Cai, Xiang-Zhen Kong

**Affiliations:** ^1^Department of Otolaryngology-Head and Neck Surgery, The First Affiliated Hospital of Zhengzhou University, Zhengzhou, 450052 Henan, China; ^2^Department of Child and Adolescent Healthcare, The Children's Hospital of Soochow University, Suzhou, Jiangsu 215021, China; ^3^Department of Pharmacy, The First Affiliated Hospital of Zhengzhou University, Zhengzhou, China; ^4^Henan Key Laboratory of Precision Clinical Pharmacy, Zhengzhou University, Zhengzhou, China

## Abstract

**Objective:**

The effects of lead exposure on cognitive function have been studied intensively over the past decade, but less attention has focused on its impact on auditory function. This study is aimed at investigating the effect of lead on the cochlea and the molecular mechanisms responsible for its actions.

**Methods:**

0.2% lead acetate was administered to rats in drinking water for 30, 60, and 90 days. Brainstem auditory evoked responses (ABR) were recorded, and morphological changes in the hair cells were observed. We also measured glutathione (GSH) and malondialdehyde (MDA) concentrations and antioxidant enzyme activities such as catalase (CAT), superoxide dismutase (SOD), glutathione peroxidase (GSH-Px), and glutathione reductase (GR) activities in the cochlea.

**Results:**

Lead exposure increased the ABR threshold and slightly prolonged the latencies of wave II and wave IV in rats. Abnormally shaped hair cells and loss of hair cells were found in the cochlea basilar membrane, together with degenerative changes in spiral ganglion neurons following lead exposure. The activities of some antioxidant enzymes were also reduced in association with upregulation of MDA expression. These effects may be caused by impaired catalytic function of the enzymes as a result of lead interaction.

**Conclusion:**

The antioxidant system of the cochlea in the immature rat brain is highly vulnerable to developmental lead exposure. Oxidative stress may therefore represent a possible mechanism for lead-induced auditory deficits.

## 1. Introduction

Lead is one of the most widespread and insidious environmental toxins and is primarily derived from human activities and from a variety of products such as paints, cosmetics, building materials, gasoline additives, and water pipes [1]. As a nonessential element for life, lead demonstrates strong neurotoxicity, inducing cognitive deficits and impaired learning and memory [2–4], as well as inducing oxidative stress [5–7]. Lead exposure was previously shown to be a high-risk factor for auditory system disturbances [8], with potentially more severe auditory implications than noise exposure [9]. Children are more vulnerable to lead toxicity than adults [10], and even blood lead levels below 100 *μ*g/L have been shown to impair both the peripheral and central portions of the auditory system in children [11, 12].

Previous studies showed that lead exposure increased hearing thresholds [13, 14], the latency of the auditory brainstem response [11, 15], and the auditory nerve action potential threshold [16]; altered the axonal integrity and myelin organization within the cochlear nerve, brainstem auditory nuclei, and white matter [16–18]; and reduced glucose metabolism in several auditory centers [19]. Lead can easily penetrate the blood-brain barrier and act directly on the neurons of the auditory center by injuring astrocytes and the endothelial microvasculature [20]. However, the impact of lead exposure on the cochlea and its mechanisms of action remain poorly understood.

Dysfunction or loss of outer hair cells (OHCs) and inner hair cells (IHCs) has been assumed to be a predominant cause of sensory hearing loss, including age-related and noise/chemical-induced hearing losses. Previous studies found that some environmental toxicants, such as manganese and mercury [21, 22], were toxic to OHCs. However, the impact of lead on hair cells has been less studied. Lead exposure has been shown to cause the generation of excessive amount of reactive oxygen species (ROS) and to alter the antioxidant defense systems in animals and in occupationally exposed workers [6].

In the current study, we exposed postweaning rats to lead to imitate conditions in children and adolescent. And we detected morphological changes, ultramicrostructures of spiral ganglion and the auditory nerve, and brainstem pathways by scanning electron microscopy, transmission electron microscopy, and auditory evoked responses (ABR), respectively. In addition, we examined the intensity of oxidative stress intensity and the activities of key antioxidative enzymes in rats with and without lead exposure. Consequently, we hypothesized that lead-induced ototoxicity was associated with the decreased activity of cochlear antioxidant enzyme, enhanced lipid peroxidation, and elevated ABR thresholds in immature rats. The results of this study will improve our understanding of lead-induced auditory function damage, explore the mechanisms responsible for these effects, and provide new clues for clinical treatment of lead poisoning.

## 2. Materials and Methods

### 2.1. Animals and Exposure to Lead

The experimental procedures were approved by the Animal Care and Use Committee of the First Affiliated Hospital of Zhengzhou University and were performed according to the guideline of the National Institutes of Health for the care and use of laboratory animals (NIH publication no. 80-23). Male postweaning Wistar rats aged 3 weeks and weighing 60–80 g each at the beginning of the experiments were used, and details of the model can be found in our earlier publications [23, 24]. The rats were housed under specific pathogen-free conditions at a constant temperature (23–28°C) and a 12 h light/dark cycle, with food and water *ad libitum*. According to previous literature [25, 26], lead-treated animals (*n* = 45) were exposed for to 0.2% lead acetate (Sigma-Aldrich, Shanghai, China) in the drinking water for 30, 60, and 90 days. An additional group of control, lead-untreated rats served as the “Day 0” group (*n* = 15). We performed hearing measurements and morphological analyses. There were no significant differences in food and water intake or body weights between lead-exposed and unexposed rats. All animals were killed at 90 days for analysis.

### 2.2. ABR Recording

ABR test was assessed in detail in previous reports [27, 28]. Each rat was deeply anesthetized with pentobarbital sodium (40 mg/kg intraperitoneal) and then placed on an electric heating plate (37.1–37.5°C) to keep them warm. All test procedures were carried out in a sound-isolated room. Within 5 min of anesthesia, the reference electrode was placed beneath the pinna of the test ear, the ground electrode beneath the apex of the nose, and the active electrode beneath the skin on the top of the head. The ABR test started immediately after the needle electrodes had been fixed. Each test ear could receive the stimulus signal through a digital signal processor (Tucker Davis, TDT RZ6, USA). Tone-burst stimuli were measured in 5 dB increments from a 90 to 10 dB sound pressure level (SPL). The threshold was obtained by identifying the lowest level of ABR wave II. The hearing threshold was defined as the lowest intensity of a pure tone that was just audible to the subject.

### 2.3. Determination of Lead Contents

Six rats from each group were used to determine the lead levels in different tissues described in detail in previous reports [23, 24]. Blood samples were obtained via the tail vein into heparinized syringes, and cerebrospinal fluid (CSF) and cochleas were placed in polyethylene tubes in 400 *μ*L nitric acid (70 g/L). The samples were digested by microwaving and cooling. After centrifugation, 150 mg cerebrospinal fluid supernatant was absorbed. The digested materials were diluted with Milli-Q water to a suitable dilution. The samples were analyzed using a Hewlett Packard HP 4500 Mass 208 inductively coupled plasma mass spectrometer (7500ce ICP-MS, Agilent Techno., USA). Analyses were done in triplicate.

### 2.4. Scanning Electron Microscopy

Rats (Day 0 and Day 90) were anesthetized deeply with 2% sodium pentobarbital and perfused through the ascending aorta with 2% glutaraldehyde using a modification of the method described by Liu et al. [29]. For scanning electron microscopy, 2% glutaraldehyde and 2% paraformaldehyde-fixed inner ears were microdissected, dehydrated stepwise in ethanol solutions, and eventually dried to a critical point. Prepared inner ears were then mounted on aluminum stubs with colloidal silver adhesive and sputter coated with gold palladium before imaging in a Hitachi S-800s scanning electron microscope (Philips, Eindhoven, North Brabant, Netherlands). The ImageJ program was used for quantitative analysis. The numbers of hair cells were measured [30].

### 2.5. Transmission Electron Microscopy

Rats (Day 0 and Day 90) were anesthetized deeply with 2% sodium pentobarbital and perfused through the ascending aorta with 2% glutaraldehyde described in detail in our earlier publication [31]. Cochleas were removed and the basal membranes were microdissected. The tissue samples were then immersed in 2% glutaraldehyde and 1% osmium tetroxide for 2 h at 4°C. The tissue blocks were routinely dehydrated using a graded ethanol series. After displacing the ethanol with propylene oxide, the tissues were embedded in Epon and consecutive, ultrathin sections were cut at 80 nm using a diamond knife. The sections were stained with lead citrate and observed under a CM-120 TEM (Philips).

### 2.6. Determination of Glutathione, Malondialdehyde, and Superoxide Dismutase

Glutathione (GSH), malondialdehyde (MDA), and enzymes (CAT, SOD, GSH-Px, and GR) were purchased from Sigma Chemicals (St. Louis, MO, USA). GSH was determined by high-pressure liquid chromatography using a modification of the method described by Fariss and Reed [32]. CAT and SOD activity was determined at room temperature according to the methods of Aebi [33] and Misra and Fridovich [34]. GSH-Px and GR activity was determined by methods of Flohe and Gunzler [35] and Carlberg and Mannervik [36] at 37°C. The extent of lipid peroxidation was estimated by the concentration of thiobarbituric acid-reactive products, measured according to Ohkawa et al. [37]. The concentrations of thiobarbituric acid-reactive products (MDA levels) were determined using 1,1,3,3-tetraethoxypropane as a standard, and the results were expressed as nmoles of MDA/mg/protein.

### 2.7. Statistical Analysis

All data were described as mean ± standard deviation (SD). *T*-test was applied to statistical analysis in most of the experiments. The data for pro- and antioxidative parameters such as GSH, CAT, SOD, GSH-Px, GR, and MDA were analyzed statistically using one-way analysis of variance followed by Duncan's multiple range test for comparison of the lead-exposed group with the control group. *p* < 0.05 was considered to indicate a statistically significant result.

## 3. Results

### 3.1. Effects of Lead Exposure on ABR Threshold

**Figure 1:**
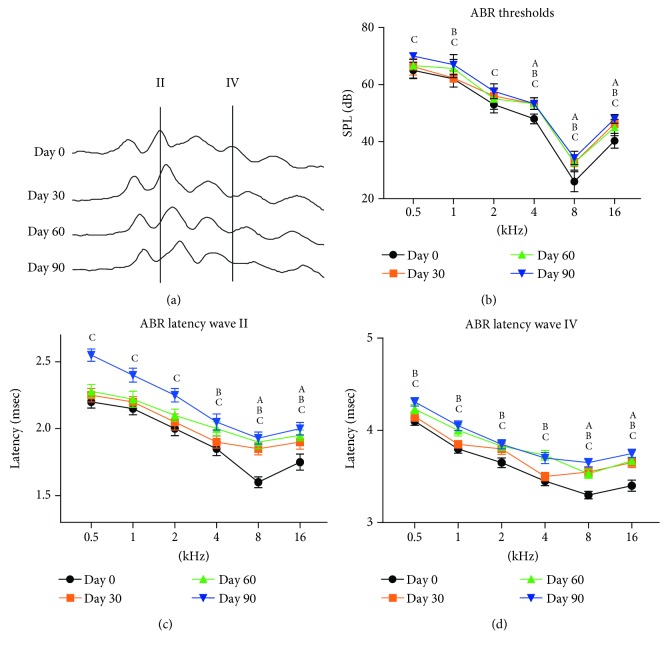
Representative ABR traces in rats exposed to lead, recording with tones of 2 kHz at an SPL of 70 dB. (a) Vertical lines indicate latencies of wave II and wave IV in controls. (b) Group mean ABR thresholds in different groups exposed to lead. ABR latencies in different groups exposed to lead for (c) wave II and (d) wave IV. Letters indicate significant difference (*p* < 0.05) between the (A) Day 0 and Day 30, (B) Day 60, and (C) Day 90 groups.

Representative ABR traces are shown in Figure 1(a) and the ABR thresholds at 0.5, 1, 2, 4, 8, and 16 kHz in control and lead-exposed rats are shown in Figure 1(b). At 0.5 kHz, ABR thresholds were only significantly elevated in the Day 90 group (*p* < 0.05), compared with control rats. At 1 kHz, ABR thresholds were significantly elevated in the Day 60 (*p* < 0.05) and Day 90 groups (*p* < 0.05), compared with control rats. At 2 kHz, ABR thresholds were only significantly elevated in the Day 90 group (*p* < 0.05). At 4, 8, and 16 kHz, ABR thresholds were significantly elevated in the Day 30 (*p* < 0.05), Day 60 (*p* < 0.05), and Day 90 groups (*p* < 0.05) compared with control rats.

### 3.2. ABR Latencies

In addition to threshold elevations, latencies were slightly prolonged in rats with developmental exposure to lead. Latencies of early wave II and wave IV were increased within the frequency range from 0.5 to 16 kHz (Figures 1(c) and 1(d)). ABR latencies of wave II at 0.5–2 kHz were significantly delayed only in the Day 90 group (*p* < 0.05), compared with control rats, while at 4 kHz, ABR latencies were significantly delayed in the Day 60 (*p* < 0.05) and Day 90 groups (*p* < 0.05). ABR latencies at 8 and 16 kHz were significantly delayed in the Day 30 (*p* < 0.05), Day 60 (*p* < 0.05), and Day 90 groups (*p* < 0.05).

ABR latencies of wave IV at 0.5–4 kHz were significantly delayed only in the Day 60 (*p* < 0.05) and Day 90 groups (*p* < 0.05) compared with control rats, while latencies at 8 and 16 kHz were significantly delayed in the Day 30 (*p* < 0.05), Day 60 (*p* < 0.05), and Day 90 groups (*p* < 0.05).

### 3.3. Accumulation of Lead in Rat Tissues following Chronic Exposure

Table 1 shows the lead concentrations in different tissues from experimental and control rats. As expected, lead levels were significantly higher in tissues from exposed rats, compared with unexposed rats (*p* < 0.05).

### 3.4. Effect of Lead Exposure on Hair Cells

**Figure 2:**
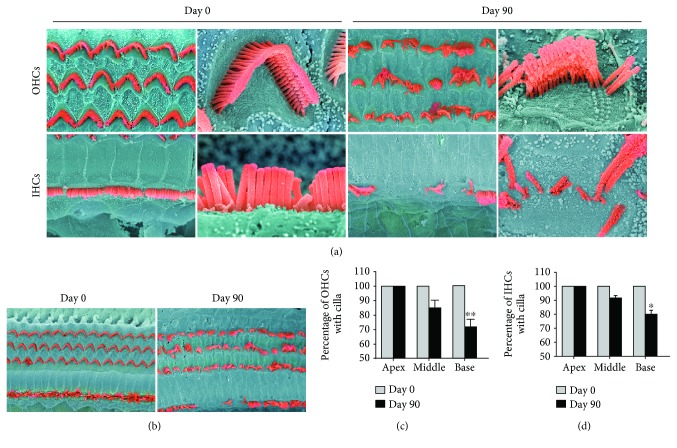
Morphological changes in hair cells following lead exposure. (a, b) Scanning electron microscope images of the representative cochlea sensory epithelium surface. Day 0 tissue displayed three rows of orderly OHCs with V-shaped stereocilia bundles. Lead exposure resulted in irregular stereocilia bundles of hair cells, and some stereocilia bundles showed a disorganized or fused shape or were lost. (c) Quantification of cilia in OHCs. (d) Quantification of cilia in IHCs. The percentages of missing cilia were calculated and compared between the two groups. Values represent mean ± SD, *n* = 3. (^∗^*p* < 0.05, ^∗∗^*p* < 0.01).

In the Day 0 group, OHCs with V-shaped stereocilia bundles were arranged in three orderly rows (Figures 2(a) and 2(b)). Compared with control rats, lead exposure resulted in an irregular arrangement of stereocilia bundles of the hair cells, with some stereocilia bundles showing a disorganized or fused shape or being lost.

### 3.5. Effect of Lead Exposure on the Ultramicrostructure of Spiral Ganglion Neurons

**Figure 3:**
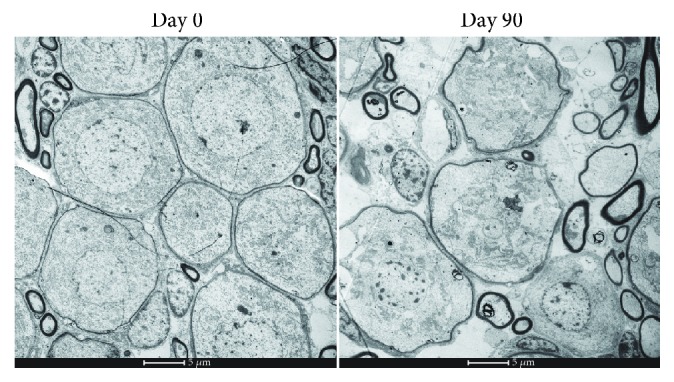
Effects of developmental lead exposure on the ultramicrostructure of spiral ganglion neurons. Spiral ganglion neurons in the control group had a round surface with a smooth cell membrane. Spiral ganglion neurons in lead-exposed rats showed a recessed membrane and loose cytoplasm with autophagosomes, indicating degenerative changes.

Spiral ganglion neurons in the control group had a rounded surface and smooth cell membrane. In contrast, spiral ganglion neurons in lead-exposed rat showed recessed membranes, loose cytoplasm, and some autophagosomes in the cytoplasm, indicative of degenerative changes (Figure 3).

### 3.6. Biochemical Data

**Figure 4:**
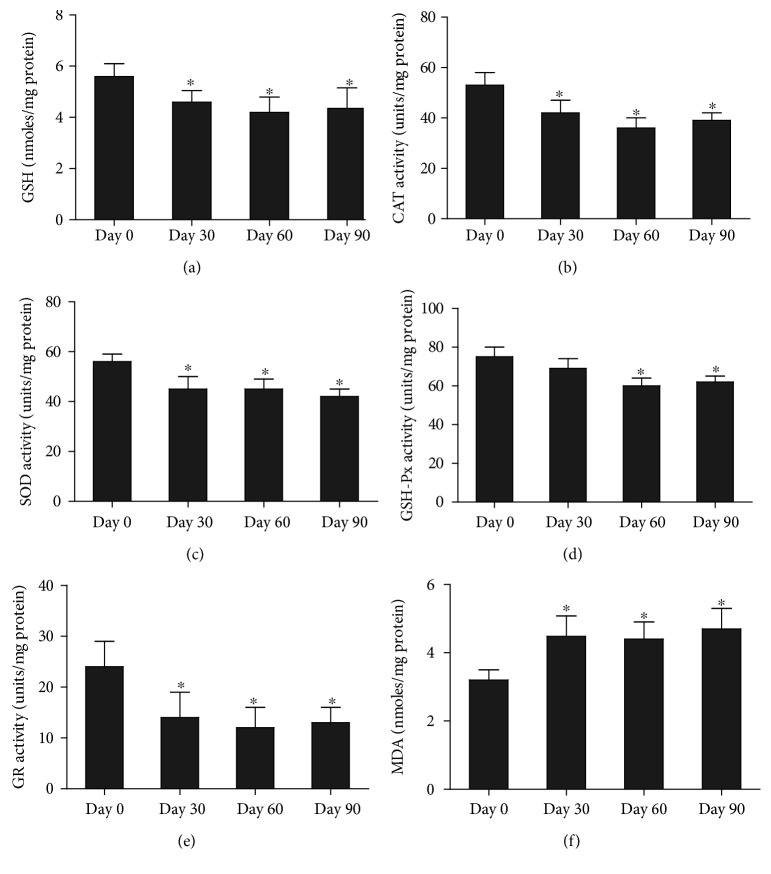
Changes in cochlea GSH content (a), CAT activity (b), SOD activity (c), GSH-Px activity (d), GR activity (e), and MDA level (index of lipid peroxidation) (f) following chronic lead exposure in rats. GSH: nmoles/mg protein; MDA: nmoles/mg protein; enzyme activities (CAT, SOD, GSH-Px, and GR) are expressed as units/mg protein. Each value represents mean ± SD of *n* = 8. (^∗^*p* < 0.05).

Lead treatment was associated with a significant reduction in GSH levels (Figure 4(a)) compared with controls (*p* < 0.05). At the same time, CAT activity (Figure 4(b)), SOD activity (Figure 4(c)), GSH-Px activity (Figure 4(d)), and GR activity (Figure 4(e)) were also significantly reduced in lead-treated rats (*p* < 0.05). In contrast, MDA levels were significantly higher in cochleas of lead-exposed rats compared with control rats (Figure 4(f)) (*p* < 0.05).

## 4. Discussion

Previous studies in humans and experimental animals clearly indicated that lead exposure could result in ototoxicity, including electrophysiological changes in the cochlea and hearing loss; however, the mechanisms underlying the toxic effects of lead on the auditory system remain largely unknown. The results of the present study clearly demonstrate that auditory function in rats was impaired following lead exposure. Furthermore, developmental lead exposure in rats disturbed the pro- and antioxidative balance in the cochlea. In our model of lead exposure, average lead levels in rat blood, CSF, and cochlea were significantly higher than levels in control animals.

The OHCs in the mammalian cochlea are an essential factor in hearing sensitivity, and death or damage of OHCs from the organ of Corti is the major cause of sensorineural hearing loss. In this study, we demonstrated that exposure of young animals to subchronic lead levels impaired auditory function, accompanied by morphological changes in OHCs. Following lead exposure, some stereocilia bundles showed a disorganized or fused shape and an irregular arrangement, indicating that their normal function was impaired. Our results demonstrated that subchronic lead exposure resulted in significant hearing loss in young rats, possibly as a result of damage to the OHCs. Further studies are needed to clarify the contribution of OHC loss to lead-induced hearing loss.

The degeneration of spiral ganglion neurons observed in this study may be related to hindered mitochondrial functions or to lead-induced oxidative stress [38]. Disruption of the balance between the production and elimination of ROS may lead to oxidative stress, which can affect the permeability of mitochondrial membranes and may in turn lead to neuronal apoptosis. As a defense strategy, mitochondria produce antioxidative molecules, such as GSH, and enzymes to eliminate ROS, including manganese SOD. It has therefore been suggested that oxidative stress may contribute to the pathogenesis of lead poisoning by disrupting the cellular pro-/antioxidant balance [39].

MDA is a product of lipid peroxidation and is commonly used as a biomarker of oxidative damage and membrane injury [40]. Accumulating evidence has shown that lead causes oxidative stress and that MDA levels are strongly correlated with lead concentration [40, 41]. In the present study, MDA levels were significantly higher in lead-treated rats compared with control rats, indicating that developmental lead exposure caused lipid peroxidation in the cochlea.

Depletion of tissue GSH can impair the cell's defense against the toxic effects of ROS and may lead to peroxidative cell injury [42]. Lead-induced ototoxicity may be caused by increased lipid peroxidation as a consequence of impaired antioxidant enzymes (CAT, SOD, GSH-Px, and GR) and depleted GSH levels. GSH and antioxidant enzyme activities in the cochlea were significantly lower in lead-exposed rats compared with unexposed rats. Other recent studies demonstrated that inhibition of antioxidant enzyme activity was associated with increased endogenous superoxide anions, hydrogen peroxide, and lipid peroxides, leading to calcium ion influx and pathologic changes in cochlea cells [43]. Impaired antioxidant enzyme activity in the cochlea may result in enhanced ROS-induced lipid peroxidation, leading to ototoxicity. The inhibition of antioxidant enzymes and depletion of GSH might also be associated with the observed increase in ABR threshold. The results of the current study showed a lead-induced decrease in antioxidant enzyme activities in the cochleas of rats exposed to lead during the developmental period. Lead-induced ototoxicity may thus be a result of increased lipid peroxidation, or a consequence of impaired antioxidant enzymes and depleted GSH levels. We conclude that the antioxidant system in the immature cochlea is highly vulnerable to lead exposure in developing rats. The administration of SOD prevented the superoxide-induced calcium influx in isolated hair cells [44]. The production of ROS as a result of lead exposure may lead to pathologic changes in acoustic transduction by modulating OHC motility, such as by changing cell shape [43], even resulting in cell death.

In summary, lead ototoxicity was associated with depletion of GSH, inhibition of antioxidant enzyme activities, and increased lipid peroxidation in the cochlea, resulting in elevated ABR thresholds and preferential high-frequency hearing loss. Lead exposure also resulted in changes in shape and loss of hair cells in the cochlea basilar membrane and degenerative changes in spiral ganglion neurons. We concluded that the antioxidant system in the cochlea of immature rat brains is highly vulnerable to developmental lead exposure, suggesting that oxidative stress may be one of the mechanisms responsible for lead-induced auditory deficits. Although children have limited opportunities to be exposed in lead and may endure more variation in their lead exposures, making that the model may not respond to the human complex environment, our study still identified the site of lesion associated with lead exposure along the auditory pathway and explored mechanisms responsible for lead-induced auditory deficits, which will provide new clues for clinical treatment of lead poisoning.

## Figures and Tables

**Table 1:** Lead concentrations in blood, CSF, and cochlea (mean ± SD).

Groups	Blood (*μ*g/L)	CSF (*μ*g/L)	CO (*μ*g/kg)
Day 0 (*n* = 8)	27.94 ± 4.28	23.70 ± 2.81	151.06 ± 22.87
Day 30 (*n* = 8)	557.35±63.38^∗∗^	45.93±18.63^∗∗^	1.66 × 10^5^ ± 1.74 × 10^4∗∗^
Day 60 (*n* = 8)	682.83±43.94^∗∗^	55.82±19.63^∗∗^	1.86 × 10^5^ ± 1.29 × 10^4∗∗^
Day 90 (*n* = 8)	760.85±53.25^∗∗^	75.05±12.63^∗∗^	1.96 × 10^5^ ± 1.67 × 10^4∗∗^

^∗∗^ *p* < 0.01 compared with the Day 0 group.

## Data Availability

The data used to support the findings of this study are available from the corresponding author upon request.

## Abbreviations

ABR: Auditory brainstem evoked responses
GSH: Glutathione
MDA: Malondialdehyde
CAT: Catalase
SOD: Superoxide dismutase
GSH-Px: Glutathione peroxidase
GR: Glutathione reductase
OHC: Outer hair cell
IHC: Inner hair cell
CSF: Cerebrospinal fluid.
